# Supplementary material for: Dietary Supplemented Curcumin Improves Meat Quality and Antioxidant Status of Intrauterine Growth Retardation Growing Pigs via Nrf2 Signal Pathway
Source: Animals (Basel). 2020 Mar 24;10(3):539. doi: 10.3390/ani10030539 (PMC7143559; doi:10.3390/ani10030539)
Supplement: Supplementary file 1 [file animals-10-00539-s001.pdf]

Table S1. Composition and nutrient level of basal diet.

Items	Content	
	26-56 d	56-115 d
Ingredient (%)		
Corn	54.50	53.00
Soybean	18.00	21.00
Soy oil	2.50	3.50
Extruded soybean	10.00	-
Fish meal	4.50	-
Dried whey	5.00	-
Glucose	2.00	-
Rice bran	-	8.50
Wheat middling	-	9.40
Dicalcium phosphate	0.80	1.00
Limestone	0.90	1.00
L-Lysine	0.35	0.18
DL-Methionine	0.10	0.12
Threonine	0.05	-
Sodium chloride	0.30	0.30
Premix[1]	1.00	2.00
Total	100	100
Nutrient levels [2]		
ME (MJ/kg)	14.40	13.93
CP (%)	20.46	16.90
Lys (%)	1.42	0.97
Met+Cys (%)	0.73	0.64
Thr (%)	0.86	0.65
Ca (%)	1.00	0.68
tP (%)	0.61	0.64

[1] Premix provided per kilogram of diet for 26-56 d: vitamin A, 5000 IU; vitamin D_3, 800 IU; vitamin E, 30 IU; vitamin K_3, 1.0 mg; biotin, 0.10 mg; folic acid, 0.3 mg; niacin, 10 mg; D-pantothenic acid, 10 mg; riboflavin, 3.6 mg; thiamine, 1.0 mg; pyridoxin, 1.5 mg; choline, 200 mg; Zn (ZnO), 2200 mg; Fe ($FeSO_4$), 125 mg; Cu ($CuSO_4{\cdot}5H_2O$),15 mg; Mn ($MnSO_4{\cdot}H_2O$), 40 mg; I (KI), 0.15 mg; Se (Na_2SeO_3), 0.25 mg. Premix provided per kilogram of diet for 56-115 d: vitamin A, 5000 IU; vitamin D_3, 800 IU; vitamin E, 30 IU; vitamin K_3, 1.0 mg; biotin, 0.10 mg; folic acid, 0.3 mg; niacin, 10 mg; D-pantothenic acid, 10 mg; riboflavin, 3.6 mg; thiamine, 1.0 mg; pyridoxin, 1.5 mg; Zn ($ZnSO_4$), 100 mg; Fe ($FeSO_4$), 100 mg; Cu ($CuSO_4$),10 mg; Mn ($MnSO_4$), 20 mg; I (KI), 0.10 mg; Se (Na_2SeO_3), 0.10 mg; multi-enzyme complex, containing phytase(10000 U), xylanase (20000 U), β-glucanaseat (2000 U); [2] Nutrient levels were calculated values.
